# Association between *BRCA1* P871L polymorphism and cancer risk: evidence from a meta-analysis

**DOI:** 10.18632/oncotarget.15739

**Published:** 2017-02-25

**Authors:** Limin Miao, Yang Yu, Yefeng Ji, Bo Zhang, Zhiyao Yuan, Yifei Du, Longbiao Zhu, Ruixia Wang, Ning Chen, Hua Yuan

**Affiliations:** ^1^ Jiangsu Key Laboratory of Oral Diseases, Nanjing Medical University, Nanjing, China; ^2^ Department of Oral and Maxillofacial Surgery, Affiliated Hospital of Stomatology, Nanjing Medical University, Nanjing, China

**Keywords:** BRCA1, polymorphism, cancer risk, meta-analysis

## Abstract

Breast cancer 1 (*BRCA1*) gene makes great contributions to the repair of DNA. The association between *BRCA1* P871L polymorphism and cancer risk has been investigated in a growing number of studies, but the conclusions are not conclusive. To obtain a comprehensive conclusion, we performed a meta-analysis of 24 studies with 13762 cases and 22388 controls. The pooled results indicated that *BRCA1* gene P871L variant decreased risk of overall cancer (homozygous model: odds ratio (OR) = 0.89, 95%confidence interval (CI) = 0.79-1.00; recessive model: OR = 0.89, 95% CI = 0.80-0.99). The stratified analysis observed decreased risk associated with *BRCA1* P871L in subgroups among Asians and high score studies, but not Caucasians or low score studies. In conclusion, despite several limitations, this meta-analysis suggested that *BRCA1* P871L genetic variation may be associated with decreased susceptibility to cancer.

## INTRODUCTION

Cancer is one of serious diseases severely endangering the human health and lives. In 2012, the world had 14.1 million new cases of cancer and 8.2 million patients died from cancer [1]. The balance between DNA damage and repair has long been recognized to be the main determinant of individual susceptibility to disease including cancer [2]. The damage of DNA often caused by environmental factors such as chemicals and certain types of radiation, which, is not repaired properly would wake the stability of the genome and lead to carcinogenesis [2]. Thus, the host DNA repair systems play pivotal roles in maintaining the stability of human genome.

DNA repair ability plays an important role in maintaining genomic stability via several pathways. The base excision repair (BER), nucleotide excision repair (NER) and DNA mismatch repair are responsible for single strand DNA damages [3]; Homologous recombination (HR) and non-homologous end joining (NHEJ) repair double-strand DNA breaks (DSBs) [4]. *BRCA1*, breast cancer 1, early onset, plays a key role in DSBs repair via HR pathway [5]. Interestingly, its involvement in other types of DNA repair has since come to light, including NER, BER, and NHEJ [5–8]. Therefore the loss or variant of *BRCA1* may contribute to instability of gene and tumorigenesis.

The non-synonymous P871L polymorphism in *BRCA1* gene lead to the amino acid substitution of leucine (Leu, L) for proline (Pro, P) at position 871, which is part of the interaction region for the recombinase RAD51, another critical protein in HR. Thus, P871L may confer cancer susceptibility. A growing number of studies have been performed to investigate its association and cancer risk; however, the conclusions are inconsistent. The discrepancies among these studies may due to the ethnic variation and relatively small sample size. To obtain a comprehensive conclusion, we conducted this meta-analysis to systematically evaluate the association between P871L and cancer risk.

## RESULTS

### Characteristics of eligible studies

**Figure 1:**
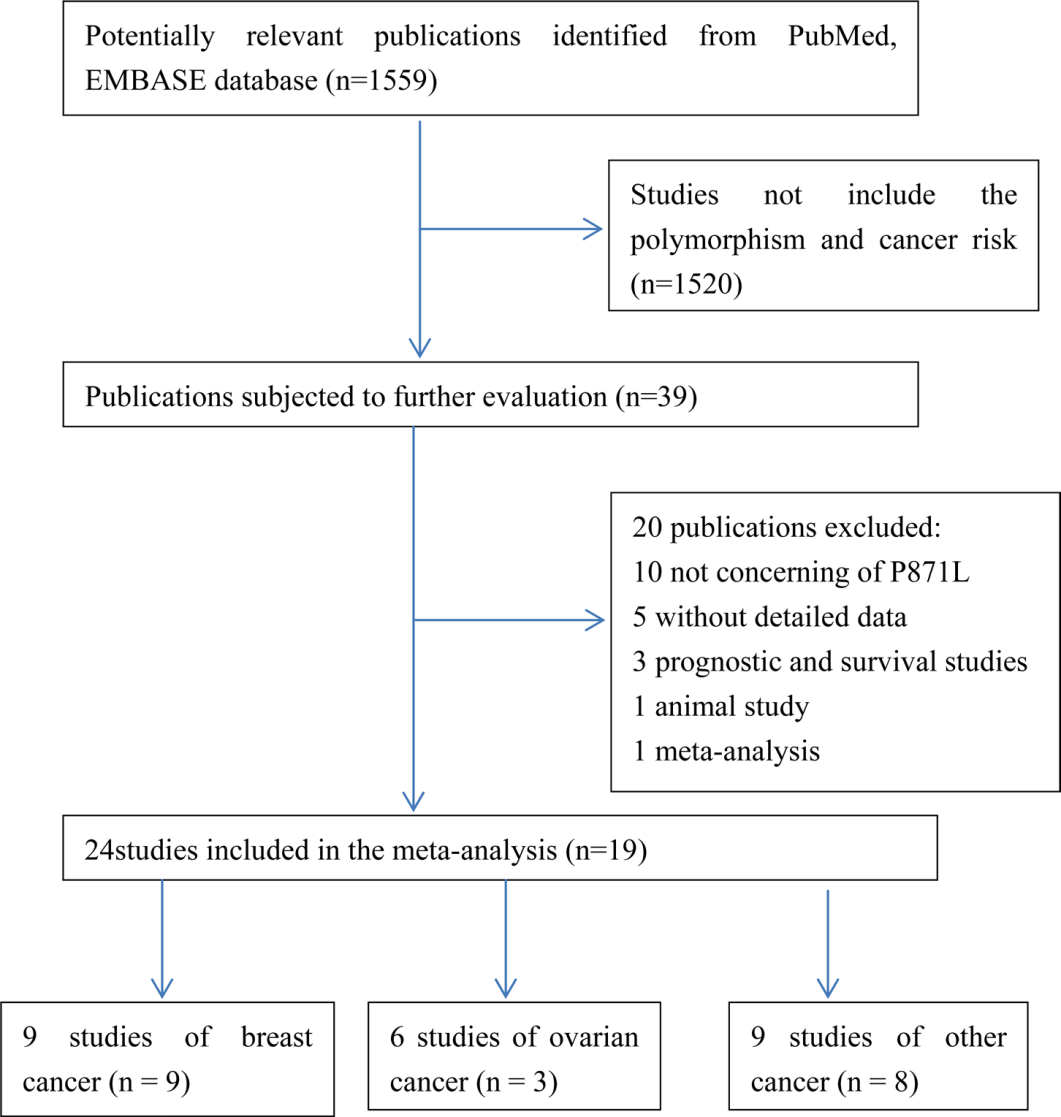
Flow chat of the study screening process in this meta-analysis

In total, 19 articles [9–27] were identified according to the inclusion and exclusion criteria, which contain 24 studies with 13762 cases and 22388 controls. The study selection process was shown in Figure 1. A total of 1559 articles were retrieved from PubMed and EMBASE. We read the title and abstract and excluded articles that are not concern about BRCA1. Then we screened out 39 articles for further evaluation. The remaining 39 articles were reviewed carefully. 20 articles were further removed, among which 10 publications not about P871L polymorphism, 5 articles without detailed data, 3 articles concerned about prognosis or survival, 1 animal study, 1 meta-analysis. Finally, 24 studies with 13762 cases and 22388 controls from 19 articles were met the inclusion and exclusion criteria in this meta-analysis. One article [11] contains two populations and another article [25] contains four populations were divided into two studies and four studies, independently. The information of these studies, including first author, year of publication, ethnicity, cancer type, number of cases and controls, HWE and quality score for each study were presented in Table 1. The distribution of genotypes in the controls of the studies was all in agreement with HWE except one study [12] due to unavailable detailed data and then only included in the dominant model.

**Table 1:** Characteristics of studies included in this meta-analysis

First author	Year	Cancer type	Ethnicity	Source of control	case	control	method	HWE	Score
Wang	2015	gastric	Asian	HB	660	800	PCR-RFLP	0.204	9
Kim	2014	NHL	Asian	PB	687	1700	PCR-RFLP	0.882	13
Zhang	2013	ESCC	Asian	PB	540	550	PCR-RFLP	0.365	12
Zhang	2013	ESCC	Asian	PB	588	600	PCR-RFLP	0.358	12
Chen	2013	NHL	Caucasian	PB	459	532	TaqMan	\	11
Hasan	2013	breast	Caucasian	HB	100	100	TaqMan	0.005	4
Wu	2013	breast	Caucasian	FB	335	408	TaqMan	0.354	7
Xu	2012	thyroid	Mixed	HB	303	511	PCR-RFLP	0.110	8
Xu	2012	salivary gland	Caucasian	HB	156	511	PCR-RFLP	0.105	9
Nicoloso	2010	breast	Caucasian	NR	247	185	PCR	0.465	4
Abbas	2010	breast	Caucasian	PB	3136	5470	MALDI-TOF MS	0.168	12
Wang	2009	breast	Asian	PB	1004	1008	PCR-RFLP	0.626	13
Zhou	2009	cervical	Asian	PB	404	404	PCR-RFLP	0.410	12
Huo	2009	breast	Asian	PB	568	624	PCR-RFLP	0.757	12
Dombernowsky	2009	breast	Caucasian	PB	1201	4119	TaqMan	0.187	10
Jeffrey	2008	Glioblastomamultiforme	Caucasian	PB	112	112	ParAllele	0.010	9
Soucek	2006	breast	Caucasian	HB	305	311	PCR-RFLP	0.820	10
Auranen	2005	ovarian	Caucasian	PB	722	830	TaqMan	0.331	9
Auranen	2005	ovarian	Caucasian	FB	310	395	TaqMan	0.900	11
Auranen	2005	ovarian	Caucasian	PB	299	781	TaqMan	0.823	10
Auranen	2005	ovarian	Caucasian	PB	297	905	TaqMan	0.333	10
Robert	2005	ovarian	Mixed	PB	305	388	TaqMan	0.141	11
Dunning	1997	breast	Caucasian	PB	801	572	ASOs hybridisation	0.805	9
Dunning	1997	ovarian	Caucasian	PB	223	572	ASOs hybridisation	0.805	7

Abbreviations: HB, hospital-based; PB, population-based; CB, center-based; FB, family based; NR, no record; MAF, minor allele frequency; HWE, Hardy-Weinberg equilibrium; ESCC, esophageal squamous cell carcinoma; NHL, non-hodgkin's lymphoma

### Meta-analysis results

**Figure 2:**
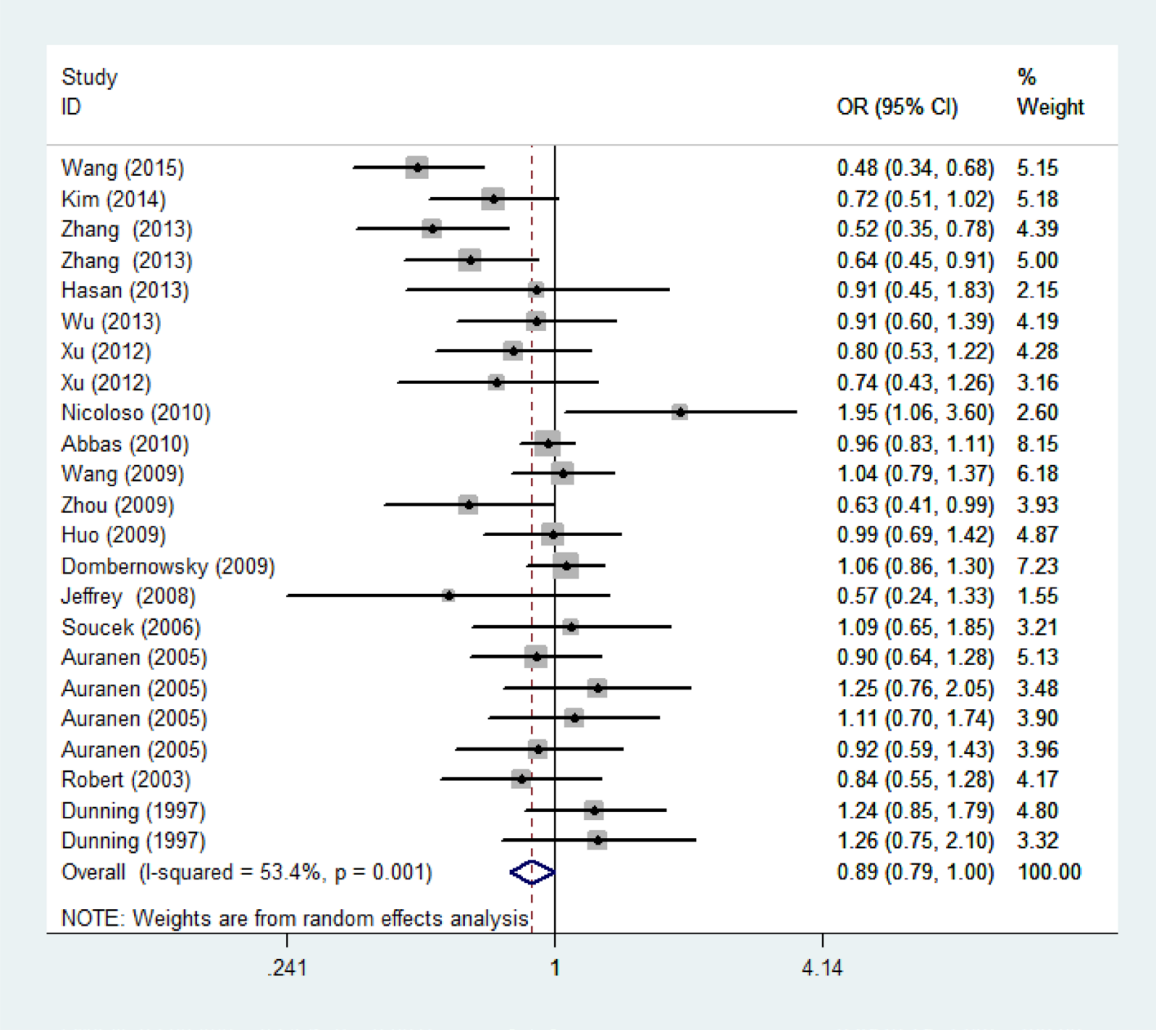
Forest plot of the association between rs799917 and overall cancer risk under homozygous model For each study, the estimation of OR and its 95% CI are plotted with a box and a horizontal line. ♢, pooled ORs and its 95% CIs.

**Figure 3:**
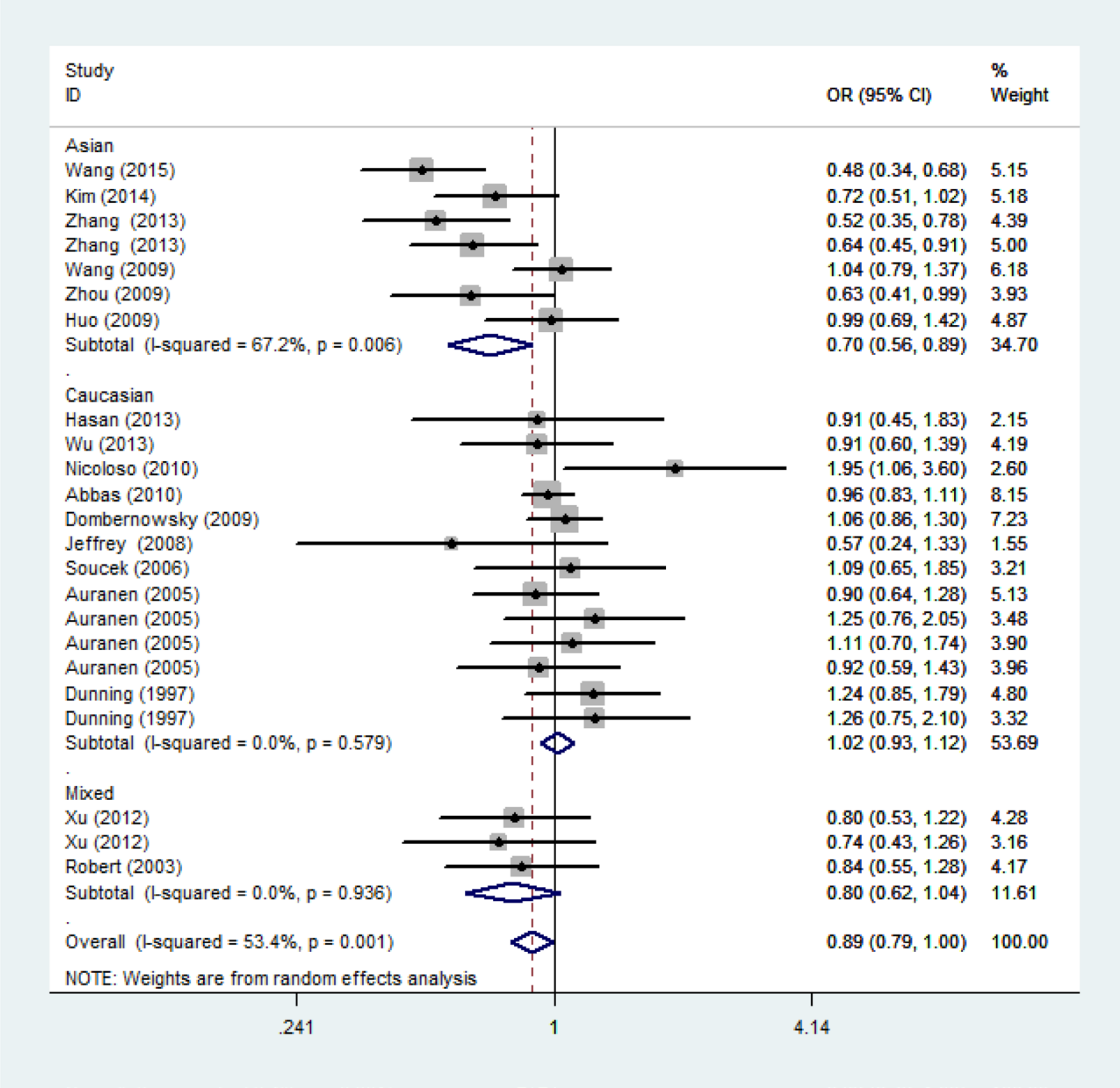
Forest plot of the association between rs799917 and cancer risk among ethnicity under homozygous model. For each study, the estimation of OR and its 95% CI are plotted with a box and a horizontal line ≢, pooled ORs and their 95% CIs.

As shown in Table 2, significant association between P871L polymorphism and overall cancer risk was observed [homozygous model: OR = 0.89, 95%CI: 0.79-1.00 (Figure 2); recessive model: OR = 0.89, 95%CI: 0.80-0.99]. Stratified analysis by ethnicity identified significant association among Asians [homozygous model: OR = 0.70, 95%CI: 0.56-0.89 (Figure 3); recessive model: OR = 0.71, 95%CI: 0.58-0.86] and mixed group (dominant model: OR = 0.81, 95%CI: 0.70-0.94; heterozygous model: OR = 0.77, 95%CI: 0.63-0.94). We further conducted the stratified analysis by control source and quality score of studies. As a result, a statistically significant association was observed in HB group (dominant model: OR=0.84, 95%CI: 0.74-0.97) and the high score group (homozygous model: OR =0.86, 95%CI: 0.75-0.97; 0.76-0.97; recessive model: OR = 0.86, 95%CI: 0.76-0.97). (Positive results were also presented in Supplemental Figure 1-8)

**Table 2:** Meta-analysis of the association between BRCA1 polymorphism and cancer risk

Variable	No. of studies ^a^	Sample size ^b^	Homozygous		Heterozygous		Dominant		Recessive	
			OR(95%CI)	P_het_^c^	OR(95%CI)	P_het_^c^	OR(95%CI)	P _het_^c^	OR(95%CI)	P_het_^c^
All	24	13762/22388	0.89(0.79-1.00)	0.001	0.99(0.93-1.06)	0.054	0.97(0.91-1.03)	0.020	0.89(0.80-0.99)	0.003
Cancer type										
Breast	9	7697/12797	1.03(0.93-1.13)	0.586	1.04(0.93-1.16)	0.032	1.04(0.95-1.15)	0.050	1.03(0.94-1.12)	0.710
Ovarian	6	2156/3871	1.00(0.84-1.19)	0.715	1.05(0.92-1.20)	0.256	1.04(0.92-1.18)	0.294	0.98(0.83-1.16)	0.832
Others ^d^	9	3909/5720	0.63(0.54-0.73)	0.604	0.90(0.82-0.99)	0.617	0.84(0.77-0.91)	0.948	0.66(0.57-0.77)	0.273
Ethnicity										
Asian	7	4451/5686	0.70(0.56-0.89)	0.006	0.98(0.90-1.07)	0.406	0.92(0.82-1.02)	0.107	0.71(0.58-0.86)	0.020
Caucasian	15	8703/15803	1.02(0.93-1.12)	0.579	1.05(0.95-1.15)	0.052	1.04(0.96-1.13)	0.106	1.02(0.94-1.11)	0.586
Mixed	2	608/899	0.80(0.62-1.04)	0.936	0.77(0.63-0.94)	0.993	0.81(0.70-0.94)	0.924	0.91(0.72-1.16)	0.933
Control source										
PB	16	11345/19167	0.89(0.79-1.01)	0.033	0.99(0.94-1.04)	0.150	0.97(0.91-1.03)	0.204	0.89(0.79-1.01)	0.015
HB	5	1524/2233	0.74(0.55-1.01)	0.085	0.90(0.78-1.03)	0.478	0.84(0.74-0.97)	0.352	0.79(0.59-1.06)	0.071
Quality score										
High	19	12554/20612	0.86(0.75-0.97)	0.002	1.00(0.93-1.07)	0.084	0.96(0.90-1.03)	0.050	0.86(0.76-0.97)	0.002
Low	5	1208/1776	1.06(0.79-1.43)	0.160	0.98(0.77-1.25)	0.093	1.01(0.78-1.30)	0.039	1.06(0.87-1.30)	0.546

^a^The number of studies included in our analysis.

^b^The number of cases and controls included in the studies.

^c^*P* value of the Q-test for heterogeneity test.

^d^Including gastric, NHL, ESCC, cervical cancer, thyroid cancer,salivary gland carcinoma and Glioblastoma multiform.

### Test of heterogeneity

Heterogeneities exist among all investigations for P871L polymorphism and overall cancer risk (homozygous: *P*_het_= 0.001; heterozygous: *P*_het_= 0.054; dominant: *P*_het_= 0.020; recessive: *P*_het_= 0.003). Thus, random-effect models were chosen to calculate the pooled ORs and corresponding 95% CIs for all genetic models. Then, a meta-regression was carried out to explore the possible source of heterogeneity by cancer type, ethnicity, control source and quality score of studies. As shown in Table 3, we found cancer type (homozygous model: *P* < 0.001; dominant model: *P* = 0.006; recessive model: *P* < 0.001) and ethnicity (recessive model: *P* = 0.017) contribute to the heterogeneity in the meta-analysis, but not the control source and quality score of studies.

**Table 3:** Meta-regression analysis of the main characteristics of the 24 studies

Study characteristics	Homozygous			Heterozygous			Dominant			Recessive		
	Coef.	95%CI	*P*	Coef.	95%CI	*P*	Coef	95%CI	*P*	Coef.	95%CI	*P*
Cancer type	−0.23	−0.32,−0.14	<0.001	−0.06	−0.14,−0.05	0.121	−0.10	−0.17,−0.03	0.006	−0.21	−0.30,−0.12	<0.001
Ethnicity	0.16	−0.03,0.36	0.094	−0.05	−0.16,−0.08	0.399	−0.02	−0.13,0.10	0.781	0.20	0.04,0.36	0.017
Control source	0.21	−0.02,0.44	0.074	0.06	−0.09,−0.20	0.444	0.95	−0.48,0.24	0.182	0.19	−0.02,0.39	0.075
Quality score	−0.22	−0.56,0.13	0.203	0.03	−0.18,−0.24	0.758	−0.03	0.24,0.19	0.787	−0.23	−0.53,0.08	0.133

### Sensitivity analysis and publication bias

**Figure 4:**
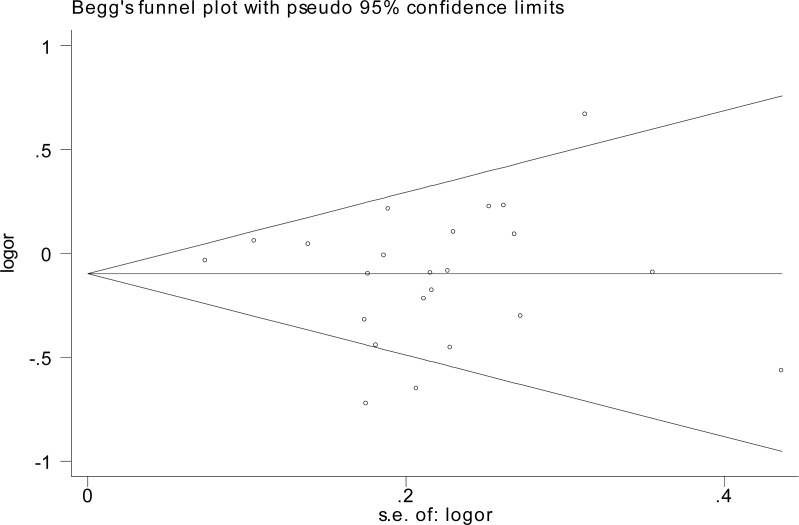
Funnel plot analysis to detect publication bias for rs799917 by homozygous model for overall analysis Each point represents a separate study for the indicated association.

The influence of each individual data on the combined OR was estimated by sensitivity analysis and no significant difference was observed in all genetic models (data were not shown). The shape of the Begg's funnel plot showed symmetric distribution in the present meta-analysis (Figure 4). The results of Egger's test were as follows: (homozygous: *P* = 0.543; heterozygous: *P* = 0.378; dominant: *P* = 0.684; recessive: *P* = 0.332), which further provided no evidence of publication bias.

## DISCUSSION

In this study, significant association between P871L polymorphism and decreased risk of overall cancer was observed in homozygous, heterozygous and recessive model. Moreover, significant association was also found in subgroups of other cancers (a combination of gastric cancer, Non-Hodgkin lymphoma, esophageal Squamous Cell Carcinoma, cervical cancer, thyroid cancer, salivary gland carcinoma and glioblastoma multiform), Asians, mixed group, as well as high quality studies. To our knowledge, this is the first time to systematically evaluate the relevance of *BRCA1* P871L polymorphism to susceptibility of overall cancer.

The *BRCA1* gene is located on chromosome17q21 and composed of 24 exons. The *BRCA1* protein plays vital roles in homologous recombination [28]. Studies also support that *BRCA1* exerts its tumor suppression function through its involvement in cell cycle checkpoint control [29, 30]. The P871L, a non-synonymous SNP is located in the coding region of *BRCA1* leads to an amino acid change from proline to leucine, is a non-conservative change as proline conveys unique structural properties to the polypeptide. However, a number of studies have reported the conflicting roles of P871L for the risk of different types of cancer. For example, P871L was indicated to be associated with risk of several cancers, such as gastric cancer, ESCC and NHL [9,11,12], but not associated with cancers including thyroid carcinoma and ovarian cancer [15,26]. And even the same kind of cancer, the results were conflicting [18,27]. A meta-analysis conducted in 2014 including 7392 cases and 12486 controls observed no significant association between P871L and breast cancer risk [31]. Our meta-analysis with more subjects found no evidence of association between P871L and breast cancer risk either. Our work found marginal association between P871Land overall cancer risk. The discrepancy between overall cancer and stratified cancer types may due to sample size, study design, carcinogenic mechanism. In addition, relevance between P871L and overall cancer risk was found in Asians but not Caucasians in stratified analysis by ethnicity, this difference may be attributed to the different genetic background and environmental exposures.

In order to make the conclusion more credible, the publication bias analysis and sensitivity analysis was conducted. Funnel plots observed no evidence of obvious publication bias. The sensitivity analysis indicated that the results are strong and no single study yield obvious effects on the pooled ORs and the corresponding CIs.

However, several limitations in this meta-analysis should be noticed. Firstly, we could not perform further subgroup analyses for certain cancer types due to the relatively small sample size. Secondly, our results were based on unadjusted assessment of ORs, which might let our results suffer from potential confounding bias. Thirdly, even though there was no publication bias, we may miss some unpublished investigations due to studies with positive results were prone to be published. Last, the heterogeneity was existed and thus we performed the random-effects model to obtain the wider CIs, which might weaken the reliability of conclusions.

In conclusion, this comprehensive meta-analysis suggests that *BRCA1* P871L polymorphism may be associated with decreased susceptibility to cancer. However, due to the limitations of the meta-analysis, well-designed, large-scale studies will be needed to confirm these findings.

## MATERIALS AND METHODS

### Search strategy

We searched the PubMed and Embase databases without language limitations for all related papers using the following terms: “*BRCA1* or P871L”, “polymorphism or variant or variation”, “cancer or carcinoma or tumor or neoplasm” (up to May 20, 2015). In addition, the references of all retrieved articles were manually searched for other related articles.

### Inclusion /exclusion criteria

Every study included in the meta-analysis is accorded with following inclusion criteria: (1) case-control studies; (2) evaluating the association between P871L polymorphism and cancer risk; (3) and sufficient information for calculating the pooled odds ratios (ORs) with 95% confidence intervals (CIs). Exclusion criteria: (1) review articles; (2) case reports, or case-only studies; (3) studies that estimated the risk of prognosis.

### Data extraction

All data were extracted from the included studies by two authors (Miao and Yu) independently. The following information was collected from each study: first author's surname, year of publication, cancer type, ethnicity, control source, sample size, genotype methods and genetic distribution of cases and controls. Cancer types were classified as breast cancer, ovarian cancer and others (“others cancer type” group including cancer subgroups that contained less than three individual studies). All subjects were categorized as Caucasian, Asian and mixed. All eligible studies were categorized as population-based (PB), hospital-based (HB) and family-based (FB) according to control sources. We assessed the quality of each study according to assessment criteria (Supplemental Table 1) and defined as high (quality score≥ 9) and low (quality score < 9) [32]. Disagreements were solved by full discussion until consensuses were reached.

### Statistical methods

Crude ORs with the corresponding 95% CIs were used to estimate the association between P871L polymorphism and cancer susceptibility. We used homozygous (TT vs. CC), heterozygous (CT vs. CC), dominant [(TT/CT) vs.CC] and recessive models [TT vs. (CC/CT)] as the models. The stratified analysis was performed by cancer type, ethnicity, control source and quality score of studies.

Z-test was used to determine the statistical significance of pooled ORs. Hardy-Weinberg equilibrium (HWE) was examined by Chi-square test based on the genotype distribution among controls. Heterogeneity was analyzed using the Chi-square based Q-test. When no heterogeneity exist (*P>*0.10 for the Q-test), the fixed effect model was performed to estimate the combined OR [33]. Otherwise, a random effect model was used to calculate the pooled OR [34]. In addition, the heterogeneity was quantified by the *I*^2^ statistics and a larger *I*^2^ value indicating a greater heterogeneity [35]. Sensitivity analysis that used to evaluate the effect of data from each study on pooled ORs was performed by sequential deleting a single study each time. Publication bias among the literatures was evaluated by Begg's funnel plot and Egger's test [36]. STATA 11.0 software (Stata Corporation, College Station, TX) was used to perform all statistical tests, all the *P* values were two-sided test and *P*< 0.05 was considered statistically significant.

## SUPPLEMENTARY MATERIALS FIGURES AND TABLE


