# Supplementary material for: Association between BRCA1 P871L polymorphism and cancer risk: evidence from a meta-analysis
Source: Oncotarget. 2017 Feb 25;8(18):30587–94. doi: 10.18632/oncotarget.15739 (PMC5444767; doi:10.18632/oncotarget.15739)
Supplement: Supplementary file 1 [file oncotarget-08-30587-s001.pdf]

Association between *BRCA1* P871L polymorphism and cancer risk: evidence from a meta-analysis

Supplementary Material

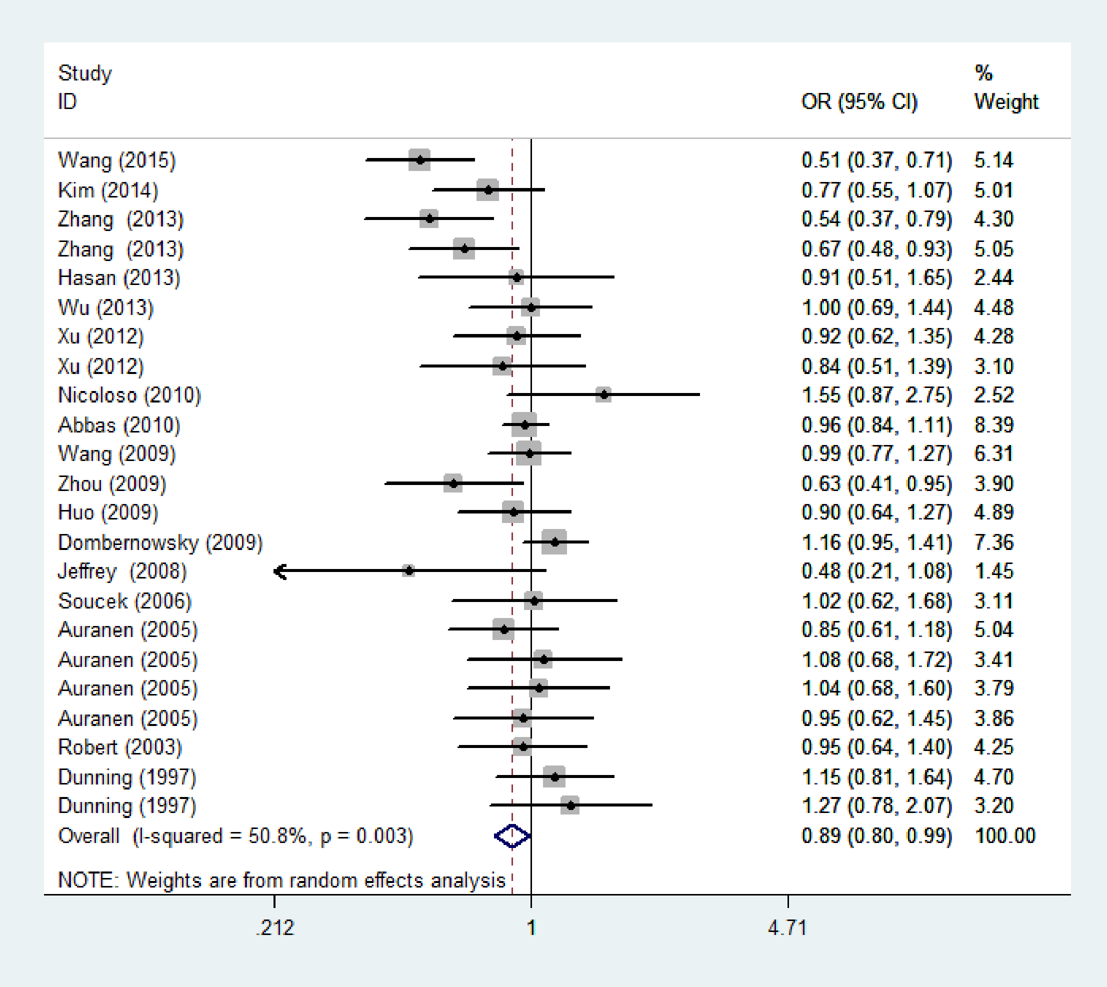

Supplemental Figure 1 Forest plot of the association between rs799917 and overall cancer risk under recessive model. For each study, the estimation of OR and its 95% CI are plotted with a box and a horizontal line.◇， pooled ORs and its 95% CIs.

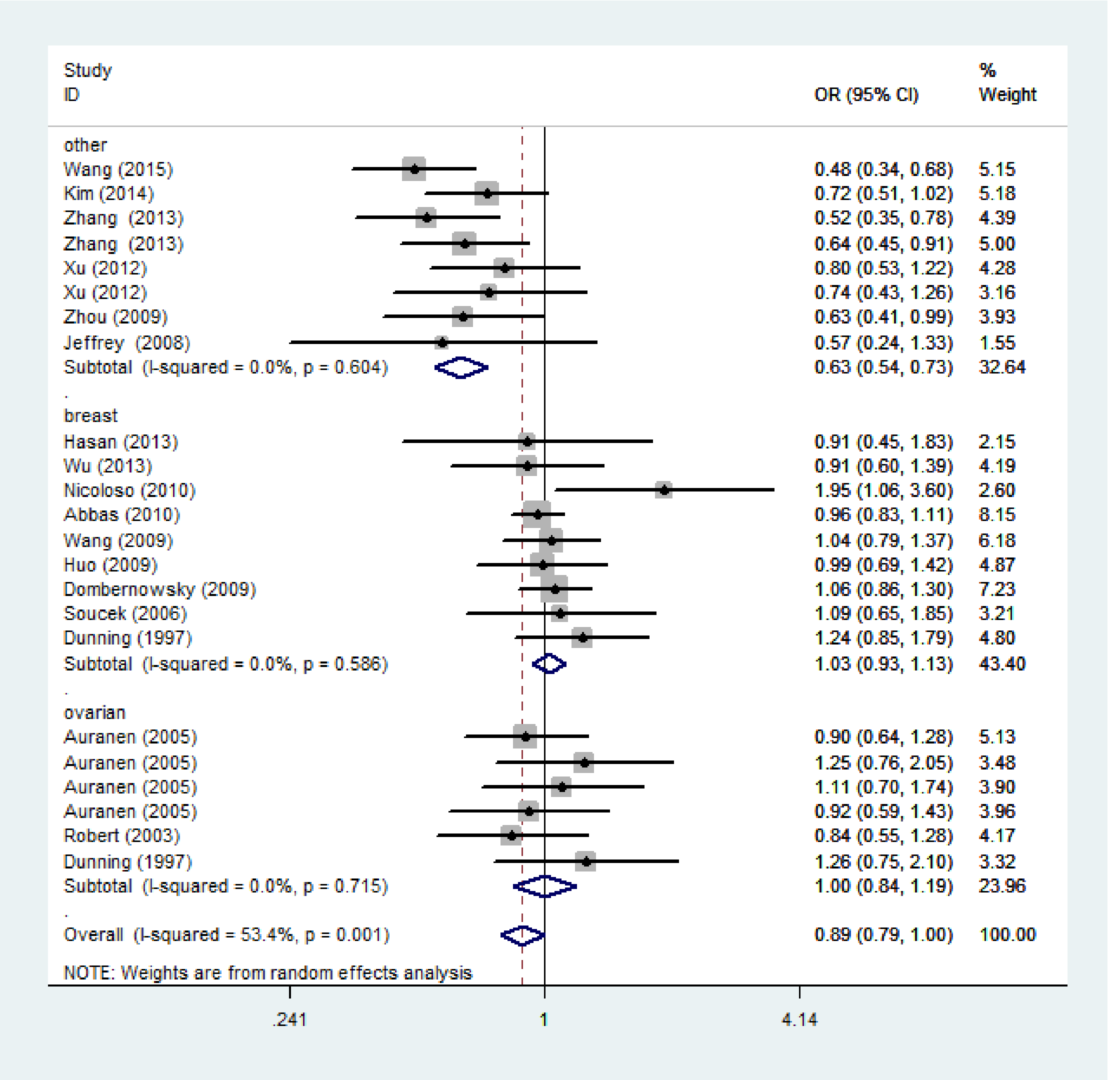

Supplemental Figure 2 Forest plot of the association between rs799917 and cancer risk among cancer types under homozygous model. For each study, the estimation of OR and its 95% CI are plotted with a box and a horizontal line. ◇， pooled ORs and its 95% CIs.

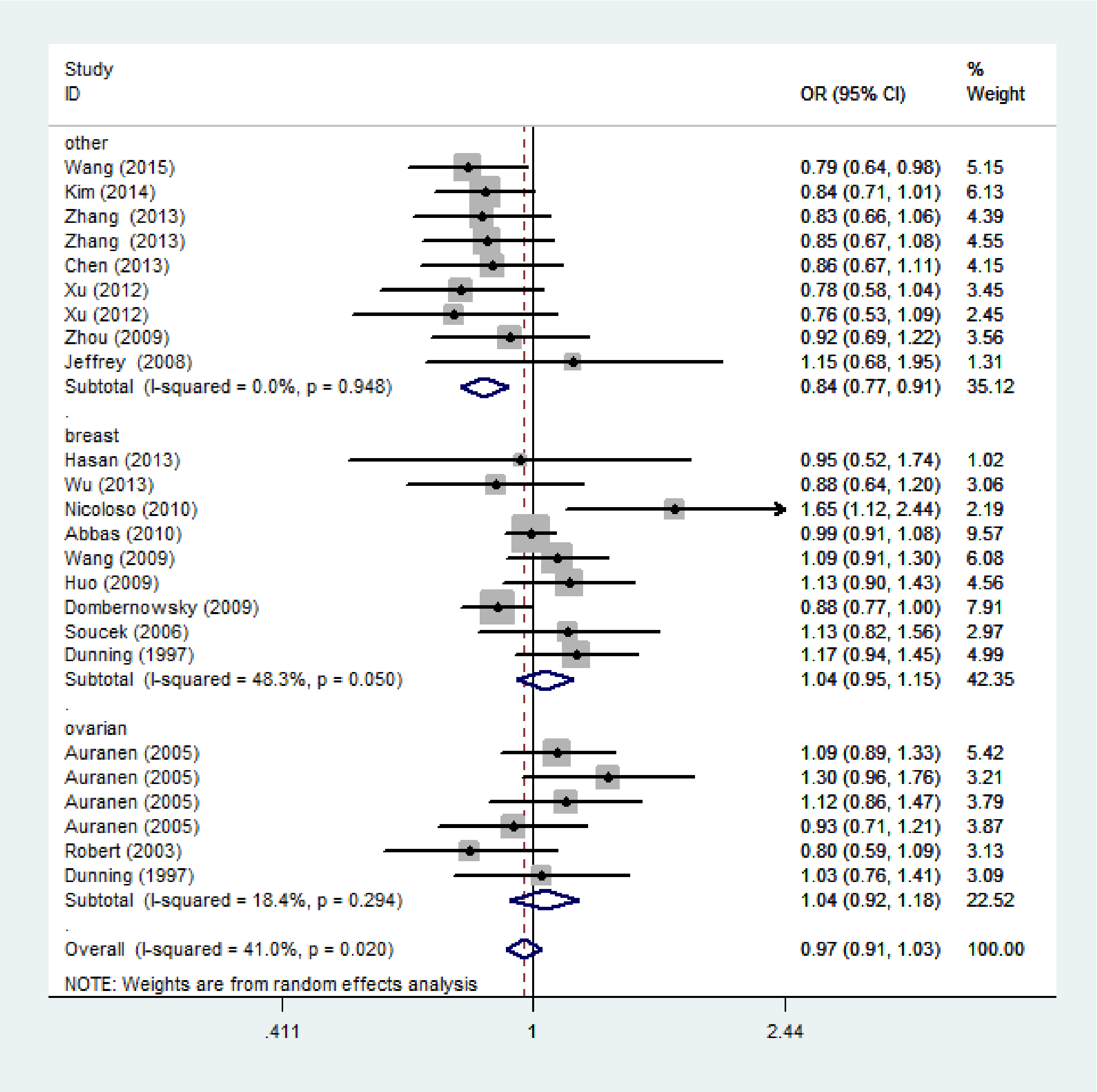

Supplemental Figure 3 Forest plot of the association between rs799917 and cancer risk among cancer types under dominant model. For each study, the estimation of OR and its 95% CI are plotted with a box and a horizontal line. ◇, pooled ORs and its 95% CIs.

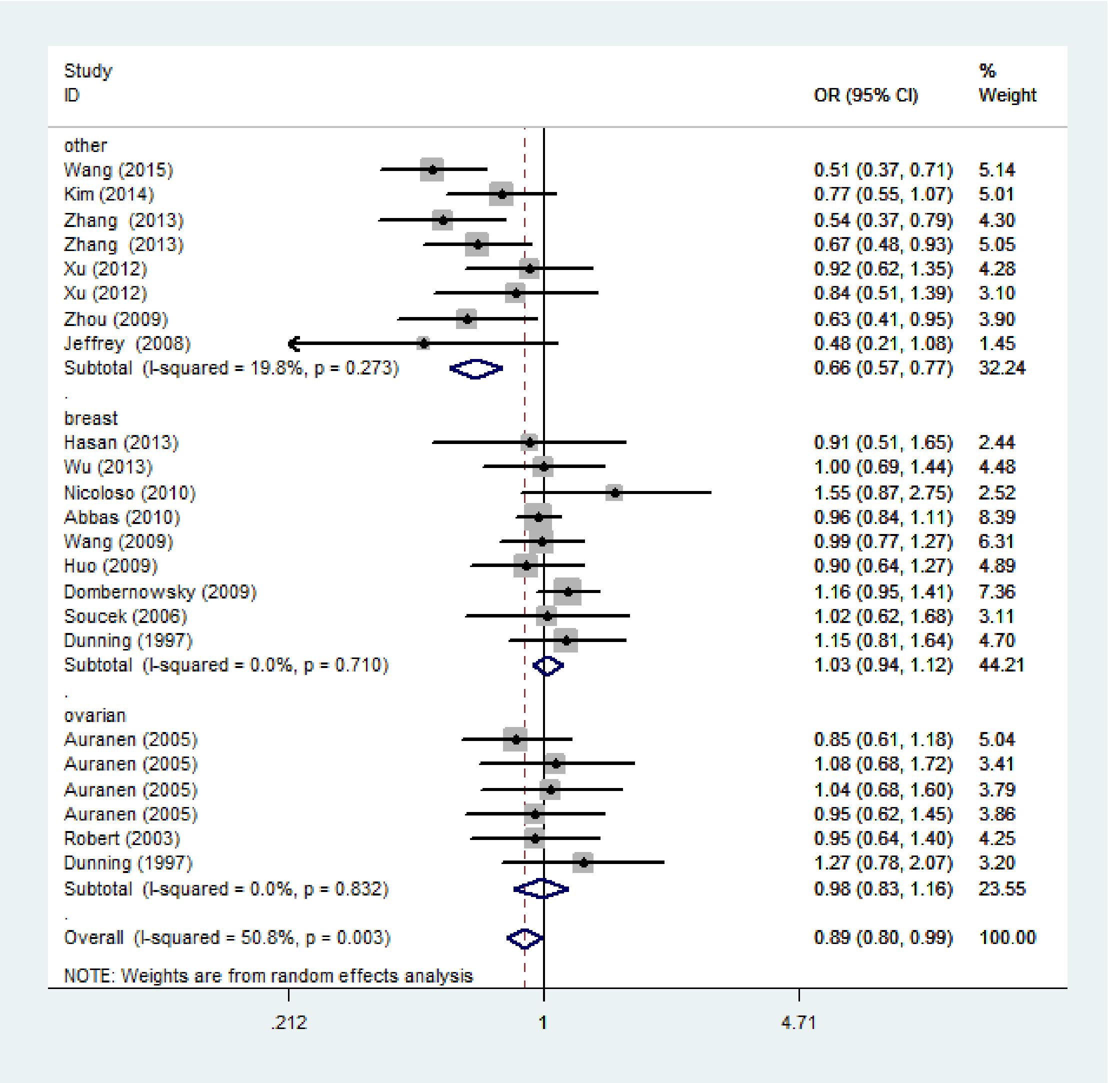

Supplemental Figure 4 Forest plot of the association between rs799917 and cancer risk among cancer types under recessive model. For each study, the estimation of OR and its 95% CI are plotted with a box and a horizontal line.◇， pooled ORs and its 95% CIs.

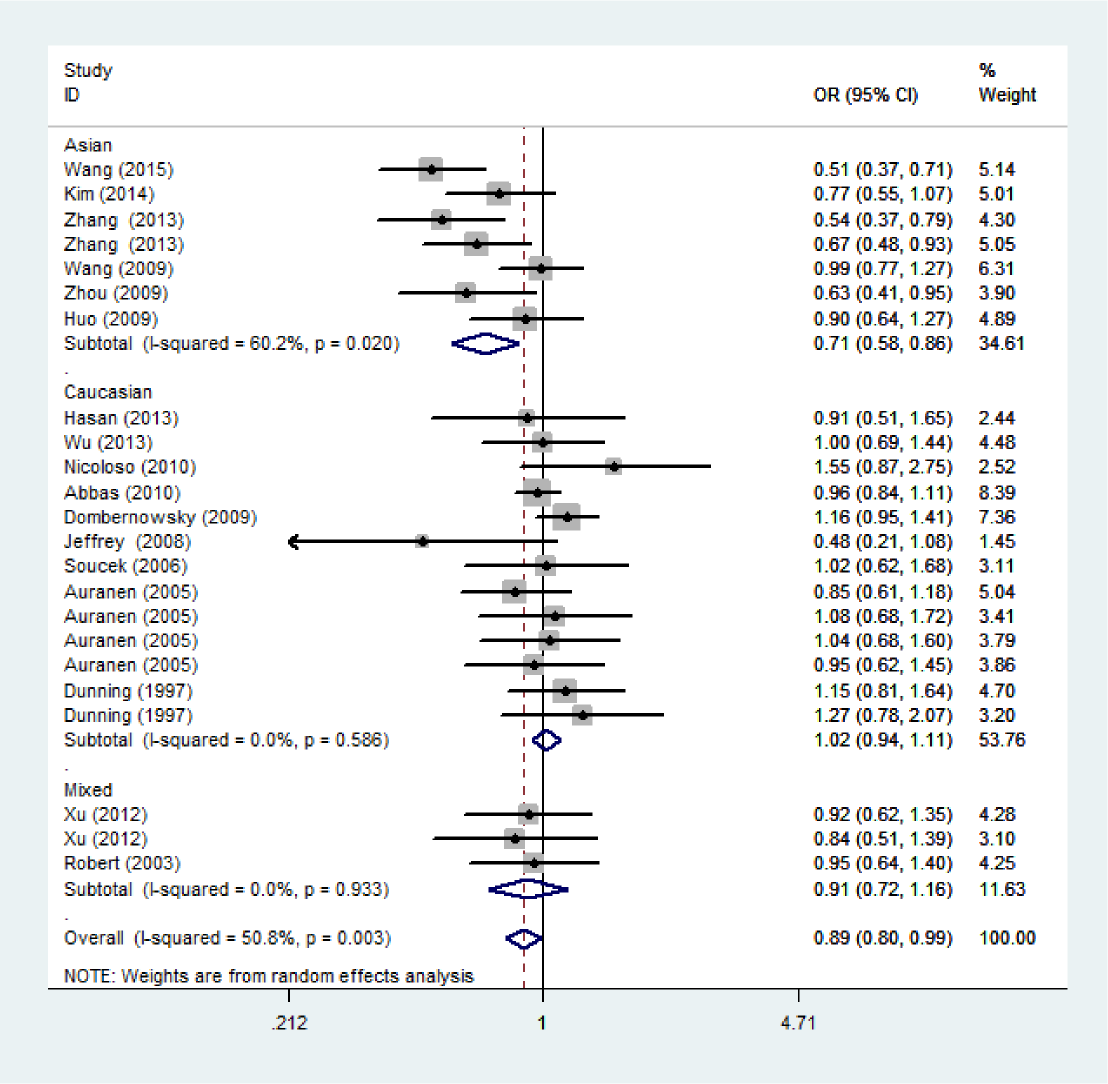

Supplemental Figure 5 Forest plot of the association between rs799917 and cancer risk among ethnicity under recessive model. For each study, the estimation of OR and its 95% CI are plotted with a box and a horizontal line. ◇， pooled ORs and its 95% CIs.

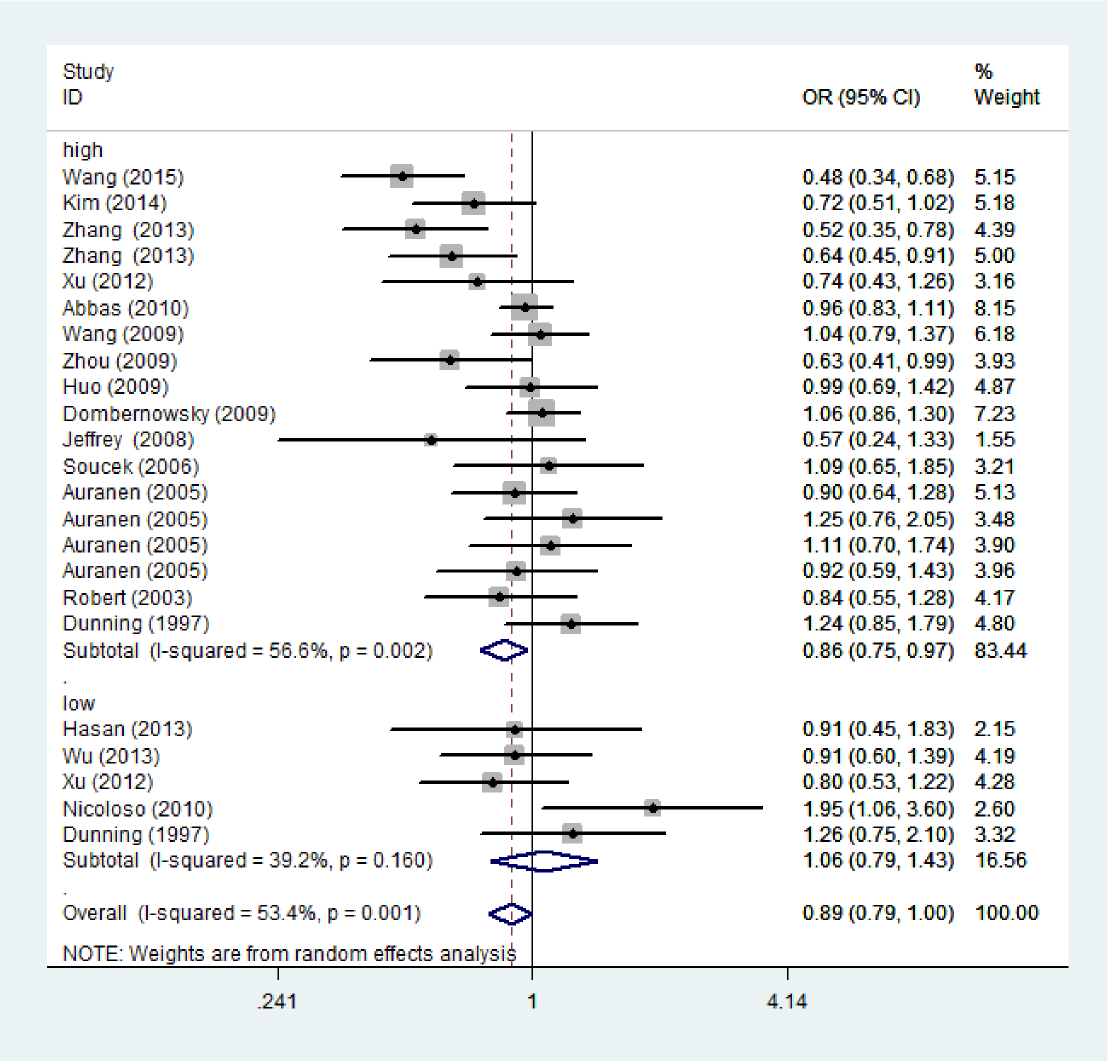

Supplemental Figure 6 Forest plot of the association between rs799917 and cancer risk among scores under homozygous model. For each study, the estimation of OR and its 95% CI are plotted with a box and a horizontal line. ◇， pooled ORs and its 95% CIs.

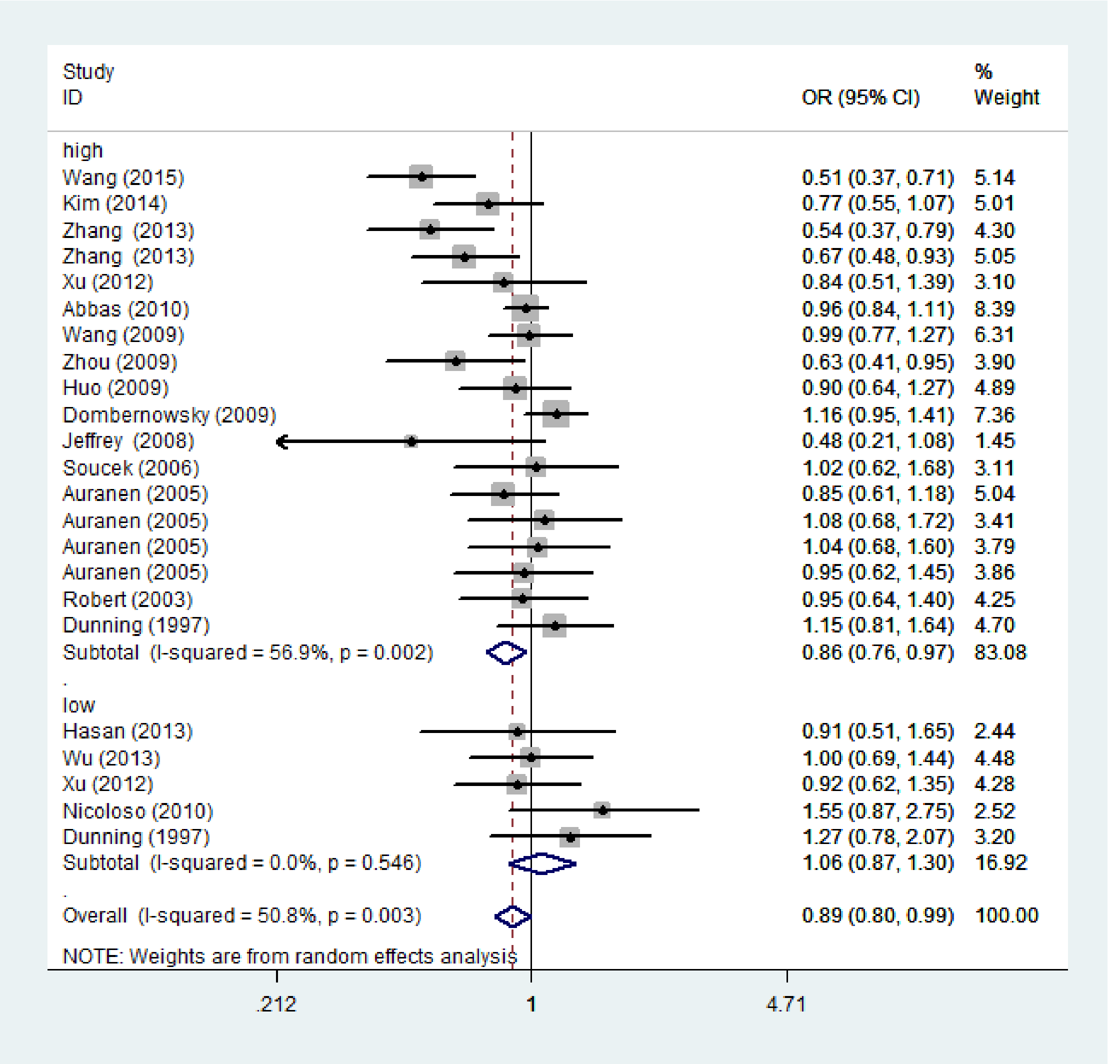

Supplemental Figure 7 Forest plot of the association between rs799917 and cancer risk among scores under recessivemodel. For each study, the estimation of OR and its 95% CI are plotted with a box and a horizontal line.◇, pooled ORs and its 95% CIs.

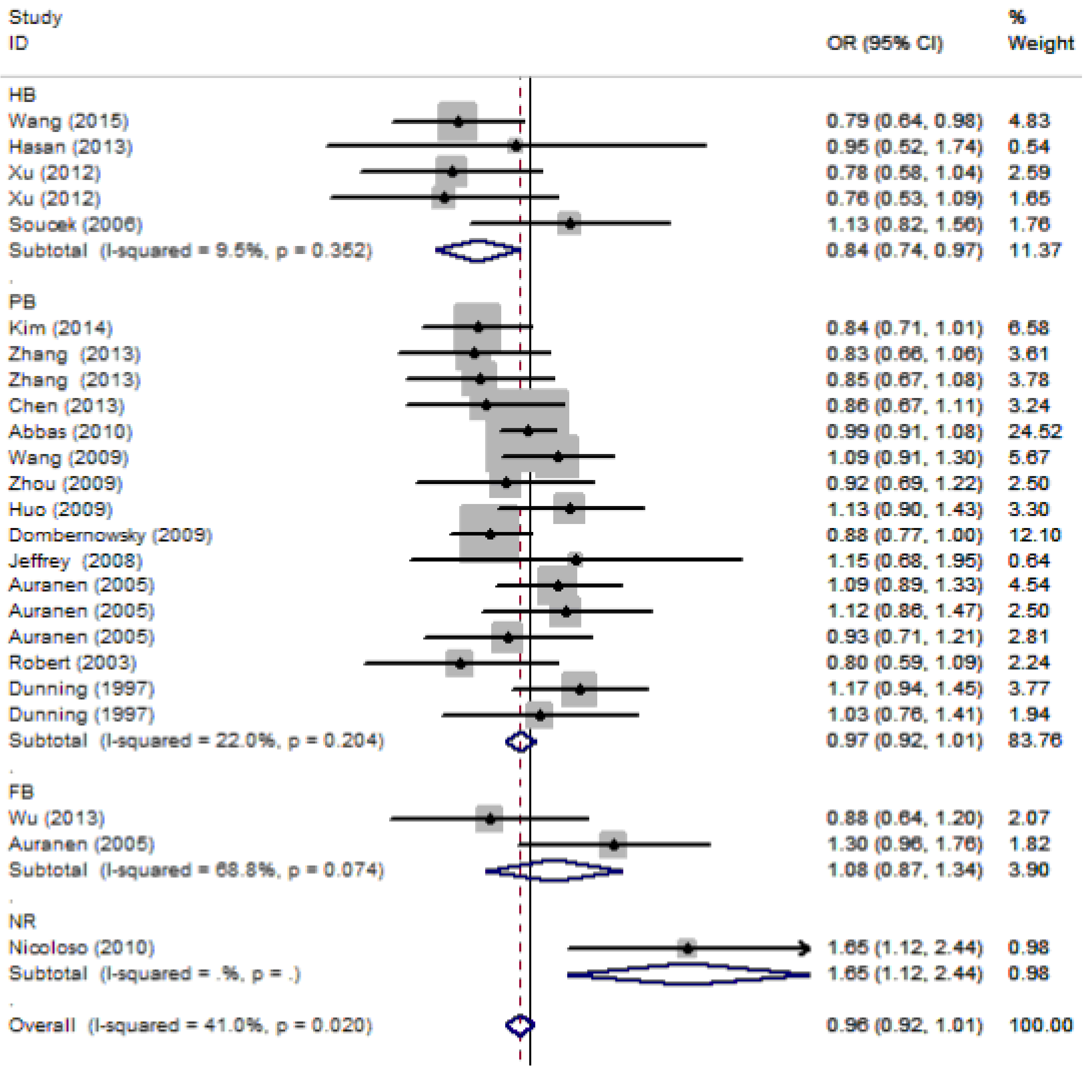

Supplemental Figure 8 Forest plot of the association between rs799917 and cancer risk among controls under dominant model. For each study, the estimation of OR and its 95% CI are plotted with a box and a horizontal line. ◇， pooled ORs and its 95% CIs.

Supplemental Table 1. Score of quality assessment

Criteria	Score
Representativeness of case	
Selected from population cancer registry	2
Selected from hospital	1
No method of selection described	0
Representativeness of control	
Population-based	3
Mixed	2
Hospital-based	1
Not described	0
Ascertainment of cancer case	
Histopathologic confirmation	2
by patient medical record	1
Not described	0
Control selection	
Controls matched with cases by age and sex	2
Controls matched with cases only by age or by sex	1
Not matched or not described	0
Genotyping examination	
Genotyping done blindly and quality control	2
Only genotyping done blindly or quality control	1
Not described	0
HWE	
HWE in the control group	1
HWD in the control group or not mentioned	0
Total sample size	
> 1000	3
501 - 1000	2
201 - 500	1
≤ 200	0
